# Reducedhumoral response against variants of concern in childhood solid cancer patients compared to adult patients and healthy children after SARS-CoV-2 vaccination

**DOI:** 10.3389/fimmu.2023.1110755

**Published:** 2023-05-25

**Authors:** Yifei Ma, Bocen Chen, Yanqi Wang, Pengfei Zhu, Nianqi Liu, Zhiying Zhang, Guanqing Zhong, Guangzhen Fu, Dao Wang, Lu Cao, Shenrui Bai, Youlong Wang, Shuqin Chen, Xiaolong Wei, Jun Lv, Ao Zhang, Xinjia Wang

**Affiliations:** ^1^ Department of Orthopedics and Spine Surgery, The Second Affiliated Hospital of Shantou University Medical College, Shantou, Guangdong, China; ^2^ Department of Bone and Soft Tissue Oncology, Cancer Hospital of Shantou University Medical College, Shantou, Guangdong, China; ^3^ Key Laboratory of Biochemistry and Molecular Biology, Hainan Medical University, Haikou, Hainan, China; ^4^ School of Public Health, Shantou University, Shantou, Guangdong Province, China; ^5^ Department of Clinical Laboratory, The First Affiliated Hospital of Zhengzhou University, Zhengzhou, Henan, China; ^6^ Faculty of Psychology, Institute of Educational Science, Huazhong University of Science and Technology, Wuhan, Hubei, China; ^7^ Department of Clinical Laboratory, State Key Laboratory of Oncology in South China, Collaborative Innovation Center for Cancer Medicine, Guangdong Key Laboratory of Nasopharyngeal Carcinoma Diagnosis and Therapy, Sun Yat-sen University Cancer Center, Guangzhou, Guangdong, China; ^8^ Department of Clinical Laboratory, The First Affiliated Hospital of Zhengzhou University, Key Clinical Laboratory of Henan Province, Zhengzhou, Henan, China; ^9^ Department of Pediatrics, The First Affiliated Hospital of Zhengzhou University, Zhengzhou, Henan, China; ^10^ Department of Hematological Oncology, State Key Laboratory of Oncology in South China, Collaborative Innovation Center for Cancer Medicine, Sun Yat-sen University Cancer Center, Guangzhou, Guangdong, China; ^11^ Department of General Surgery, Hainan Hospital of People's Liberation Army General Hospital, Sanya, Hainan, China; ^12^ Department of Pathology, Cancer Hospital of Shantou University Medical College, Shantou, Guangdong, China; ^13^ Department of Infectious Diseases and Hepatology, The First Affiliated Hospital of Zhengzhou University, Zhengzhou, Henan, China

**Keywords:** SARS-CoV-2, childhood cancer patients, variants of concern, immune response, propensity score matching

## Abstract

**Introduction:**

Although there is extended research on the response to severe acute respiratory syndrome coronavirus 2 (SARS-CoV-2) vaccines in adult cancer patients (ACP), the immunogenicity to the variants of concern (VOCs) in childhood cancer patients (CCP) and safety profiles are now little known.

**Methods:**

A prospective, multi-center cohort study was performed by recruiting children with a solid cancer diagnosis and childhood healthy control (CHC) to receive standard two-dose SARS-CoV-2 vaccines. An independent ACP group was included to match CCP in treatment history. Humoral response to six variants was performed and adverse events were followed up 3 months after vaccination. Responses to variants were compared with ACP and CHC by means of propensity score-matched (PSM) analysis.

**Results:**

The analysis included 111 CCP (27.2%, median age of 8, quartile 5.5–15 years), 134 CHC (32.8%), and 163 ACP (40.0%), for a total 408 patients. Pathology included carcinoma, neural tumors, sarcoma, and germ cell tumors. Median chemotherapy time was 7 (quartile, 5–11) months. In PSM sample pairs, the humoral response of CCP against variants was significantly decreased, and serology titers (281.8 ± 315.5 U/ml) were reduced, as compared to ACP (*p*< 0.01 for the rate of neutralization rate against each variant) and CHC (*p*< 0.01 for the rate of neutralization against each variant) groups. Chemotherapy time and age (Pearson *r* ≥ 0.8 for all variants) were associated with the humoral response against VOCs of the CHC group. In the CCP group, less than grade II adverse events were observed, including 32 patients with local reactions, and 29 patients had systemic adverse events, including fever (*n* = 9), rash (*n* = 20), headache (*n* = 3), fatigue (*n* = 11), and myalgia (*n* = 15). All reactions were well-managed medically.

**Conclusions:**

The humoral response against VOCs after the CoronaVac vaccination in CCP was moderately impaired although the vaccine was safe. Age and chemotherapy time seem to be the primary reason for poor response and low serology levels.

## Introduction

Currently, vaccines against severe acute respiratory syndrome coronavirus 2 (SARS-CoV-2) are being developed as an effective measure against the pandemic (1, 2). Tolerability, safety, and immunogenicity in vaccination for healthy children have been evidenced to be similar to results in vaccine trials of adults and proved effective in building herd immunity (3). Although children who contracted coronavirus disease 2019 (COVID-19) were shown to manifest less severe courses of disease than adults, those with immunocompromised statuses, such as cancers, are still highly vulnerable to virus infection due to altered immune status and care provided (4, 5). Thus, it is pivotal to decrease the risk of COVID-19 contraction in such populations by vaccination if proven effective and safe (6). For adult cancer patients (ACP), prospective trials have demonstrated that various types of vaccines could induce satisfactory immunogenicity without serious adverse events (7–9). Thus, current opinions from a number of organizations have unequivocally recommended cancer patients as the most prioritized group to receive vaccination (8).

Until now, there have been reports of vaccine safety or effectiveness reports in pediatric patients with blood cancer (10), with relatively good tolerability and effectiveness. However, there is a paucity of data concerning SARS-CoV-2 vaccination in the population of childhood solid cancer patients (CCP), probably due to the low incidence in the childhood population (11, 12). Evidence from non-SARS-CoV-2 vaccines may suggest comparable results in terms of safety and immunogenicity (including influenza and pneumococcal vaccines) (13). Studies found sound immunogenicity and safety profiles of such vaccines previously, and immunomodulators in therapeutic regimens may substantially alter seroconversion rates (14, 15). Results can be translated into SARS-CoV-2 vaccination for CCP but direct evidence is still needed in the population. Even though the pathology state may be different, treatment profiles have been shown to substantially influence vaccination outcomes, including chemotherapy common to all solid cancer patients. Additionally, risk factors that contribute to humoral response in CCP should be illustrated and results may be different from those of the adult population, in whom age and immune status were shown to jointly affect outcomes (9). As these questions have seldom been addressed as of now, an observational investigation into the use of inactivated vaccines in this population may provide real-world evidence for future trials.

In this pilot prospective study, we aim to compare humoral response and safety profiles between CCP and healthy children and then between CCP and adult patients.

## Methods

### Participant enrollment and study design

The multi-center, prospective study recruited CCP and childhood healthy control (CHC) to receive the standard dose SARS-CoV-2 inactivated vaccines (CoronaVac, 4 μg per 0.5 ml per shot) from the pediatric hematology/oncology cancer database (pediatric Vacan cohort, Shan 2021-137) of the five tertiary hospitals (see **Supplementary Methods**) between 13 December 2021 and 18 July 2022. An independent group of ACP was also retrospectively included to match the CCP by cancer-related treatment and time of receiving vaccines from 1 March 2021 to 1 July 2022. Ethical approval has been gained at the Second Affiliated Hospital of Shantou University Medical College (SAHSU). The study was performed according to the ethical principles of the Declaration of Helsinki and Good Clinical Practice, and all parents of the CCP and CHC provided informed consent before participation.

Inclusion criteria for CCP were as follows (1): age between 3 and 18 years old (2); diagnosis of solid cancers in the past 3 years, irrespective of the current status of disease activities or treatment; and (3) willingness to and ability to have the clinical samples tested by the researchers, including blood, feces, and urine. Key exclusion criteria were as follows (1): contraindications to receiving vaccines, including hypersensitive reactions to the adjuvants of the CoronaVac vaccines (2); the prognosis of the CCP was less than 6 months or equivalent to hospice settings (3); history of severe autoimmune, genetic, or hematological diseases (4); prior infection of SARS-CoV-2 (5); diagnosis of hematological malignancies (which will be published elsewhere); and (6) participants with positive SARS-CoV-2 antibody titers before receiving vaccines.

### Participant data

The bio-specimen in the study were per-protocol blood and routine samples of serum from clinical settings collected for downstream analysis. All participant data and samples were de-identified and stored with the analytic ID number in the current investigation. The electronic data capture systems of the SAHSU were applied to save and monitor the de-identified profiles.

The anti-SARS-CoV-2 antibody serology testing was performed prior to receiving vaccines, and 3 months after the second dose, respectively. After collection of the whole blood in Ethylene Diamine Tetraacetic Acid (EDTA) tubes, the serum was carefully collected and centrifuged for 10 min and stored at −80°C until testing. Times of blood drawing, processing, and refrigeration were recorded for each sample. The time from blood drawing to refrigeration was kept to be less than 24 h, and the process was done by the lab technicians blinded to the data. The sera were analyzed in the third-party laboratory in Guangzhou Province for antibody testing. For qualitative and quantitative analysis, the S-specific immunoglobulin G (IgG) was tested using the chemiluminescence kit (Bioscience Technology, Guangzhou, China).

Demographic and therapeutic data were extracted from the Case Record Form of the five tertiary referral hospitals. The demographic information included the gender and the age of participants (CCP, CHC, and ACP). The nutrition status of the CCP was defined with the body mass index of the corresponding growth milestones of the Chinese children and was thus divided into underweight, normal, and overweight by the treating oncologists blinded to the participant data. In the ACP group, the nutrition status was defined by the treating oncologists at follow-up. The treatment details included the cumulative chemotherapy time, steroid use history, tyrosine kinase inhibitor use, and radiotherapy history. The survival time was calculated from the time of cancer diagnosis to the time of receiving vaccines, and the chemotherapy status was also recorded as active or inactive. The pathological types of the CCP and ACP were determined by both histological and radiological evidence from the patient database of the five hospitals.

Included CCP participants received standard, two-dose CoronaVac SARS-CoV-2 vaccines at registered vaccination sites of the Centers for Disease Control and Prevention, with an intervening period of 5 to 7 weeks between the two doses. Participants with severe adverse events after the first doses of SARS-CoV-2 vaccines would not receive the second dose as required by the institutional board.

The primary outcome of the study was to report humoral responses against variants of concern (VOCs) after the COVID-19 vaccination in each group. The secondary outcome was to illustrate follow-up adverse events, or safety, of CCP during 3 months following SARS-CoV-2 vaccination. Solicited adverse events were recorded according to China National Medical Products Administration guidelines (16). Both efficacy and safety outcomes would be compared with ACP and CHC groups by propensity score-matched (PSM) analysis to minimize selection bias that may occur during the study process. Participant data, serology, and neutralization test methods are shown in supplementary details (see **Supplementary Methods**).

### Statistics and analytic protocols

The primary research goal was the humoral response against each variant, including serology conversion, serology conversion rate, and neutralization ability. The secondary goal was adverse events, including local and systemic events graded according to Common Terminology Criteria for Adverse Events. Categorical variables were recorded as numbers and percentages, and continuous variables were recorded as means ± standard deviations (SD) and median (25th–75th quartile). The power (1 − *β*) of each statistical test was calculated according to the sample size on the PASS software (V.15.0), and each test was based on a pre-specified statistical hypothesis with a type I error of 0.05.

To minimize potential bias, the propensity scores were calculated in the CCP, ACP, and CHC groups. The score was calculated with a logistic conditional regression model (17). Variables included in the regression model included treatment details and baseline demographics. For comparison between CCP and CHC groups, variables included gender and age. After calculation, the nearest neighbor head-to-head (1:1) method was applied to match each comparable participant with the upper acceptable caliper width of 0.2 without replacement (18). To find potential imbalance and to evaluate matching performance, standardized mean difference (SMD) was calculated in both unmatched and matched data (19, 20). According to Austin et al., an SMD over (√((n1+n2)/(n1*n2)))*1.96 is regarded as imbalanced pairs in the matched samples, where *n*1 and *n*2 stand for sample sizes of matching pairs (21). Thus, in matched samples, statistical tests of difference were McNemar test for categorical variables and Wilcoxon signed-rank test for continuous variables (22). Statistics used were carried out in SPSS V.23.0 software.

## Results

### Baseline characteristics

The study enrolled 121 CCP and 153 gender-matched CHC in a prospective cohort. No participant had ever received SARS-CoV-2 vaccines or been infected by SARS-CoV-2 vaccines, and all patients had negative antibody titers at recruitment. Ten CCP and 19 CHC did not finish the second dose due to concerns about future adverse events. Thus, a total of 111 CCP (73 male and 38 female patients) and 134 CHC (82 male and 52 female patients) were included in the final analysis (**Table 1**). A total of 163 (88 male and 75 female patients) ACP were enrolled that match the treatment and vaccination details of CCP (**Supplementary Table 1**). The median age of CCP was 8 (quartile, 5.5–15), and the median age of CHC was 9.5 (quartile, 6–13).

**Table 1:** Baseline characteristics of the enrolled children.

Factors		CCP *N* = 111	CHC *N* = 134	*p*-value
Age (years)	Median (quartile)	8 (5.5–15)	9.5 (6–13)	0.53
	Mean (SD)	10.2 (5.1)	9.8 (4.3)	
Blood drawing time since receiving vaccines (days)	Median (quartile)	19 (17–22)	20 (16–23)	0.97
	Mean (SD)	19.48 (3.24)	19.49 (3.75)	
Gender	Male (%)	73 (65.7)	82 (61.2)	0.46
	Female (%)	38 (34.3)	52 (38.8)	
Nutrition status	Underweight (%)	28 (25.2)	—	
	Normal for age (%)	37 (33.3)	—	
	Overweight (%)	56 (41.5)	—	
Chemotherapy status	Active (%)	20 (18.0)	—	
	Inactive (%)	91 (82.0)	—	
Steroid therapy history	Yes (%)	26 (23.3)	—	
	No (%)	85 (76.7)	—	
Chemotherapy time (years)	<0.5 years (%)	46 (41.4)	—	
	0.5 to 1 year (%)	44 (39.6)	—	
	1–2 years (%)	21 (19.0)	—	
Radiotherapy history	Naive or Never (%)	33 (29.7)	—	
	Yes (%)	78 (70.3)	—	
Pathology	PNET (%)	10 (9.0)	—	
	Neuroblastoma (%)	30 (27.0)	—	
	Nasopharyngeal carcinoma (%)	26 (23.4)	—	
	Rhabdomyosarcoma (%)	10 (9.0)	—	
	Osteosarcoma/Ewing sarcoma (%)	21 (18.9)	—	
	Hepatic blastoma (%)	4 (3.6)	—	
	Germ cell tumors (%)	10 (9.0)	—	
TKI therapy history	Yes (%)	14 (12.6)	—	
	No (%)	97 (87.4)	—	
Survival since diagnosis (months)	Median (quartile)	27 (20–37)	—	
	Mean (SD)	27.7 (21.9)	—	

CCP, childhood cancer patients; CHC, childhood healthy control; PNET, primitive neuroectodermal tumors; SD, standard deviation; TKI, tyrosine kinase inhibitors. —, Not Applicable.

As for pathology types of CCP, 10 CCP (9.0%) had primitive neuroectodermal tumors (PNET), 30 CCP (27.0%) had neuroblastoma, 26 CCP (27.0%) had nasopharyngeal carcinoma, 10 CCP (9.0%) had rhabdomyosarcoma, 21 CCP (18.9%) had osteosarcoma or Ewing sarcoma, 4 CCP (3.6%) had hepatic blastoma, and 10 CCP (9.0%) had germ cell tumors.

Forty-six CCP (41.4%) received chemotherapy for less than 6 months, 21 patients (19.0%) over 1 year, and 44 patients (39.6%) between 6 months and a year. Median chemotherapy time was 7 (quartile, 5–11) months. Twenty CCP were on active chemotherapy when vaccinated. Seventy-eight patients (70.3%) had received radiotherapy in the past year, and 26 patients (23.3%) had received steroid therapies during the past 6 months. The median time of survival of the CCP was 27 (quartile, 20–37) months.

### The humoral response was lower in CCP, with younger age combining chemotherapy associated with outcomes

Overall, after receiving two-dose vaccines, 76 of 111 CCP (68.4%) were seropositive and 35 patients had a negative response. Mean antibody titers were 281.8 ± 315.5 U/ml, and 43 (38.7%) participants had antibody levels over 300 U/ml. Univariate analysis of seroconversion failure showed a significant association with the following variables: age, pathology types, nutrition status, and chemotherapy time. Multivariate regression identified the following two variables to be independently significant (**Table 2**): age (OR = 0.84, 95% CI = 0.71–0.98, *p* = 0.03) and chemotherapy time (OR = 3.70, 95% CI = 1.71–7.98, *p*< 0.01). To quantify risk prediction of the serology failure, a nomogram was built that incorporated the two variables (**Supplementary Figure 1A**). A calibration curve was drawn to compare the actual and predicted risk of seroconversion failure (**Supplementary Figure 1B**). The concordance index of the nomogram was 0.79.

**Table 2:** Regression analysis for serology response rate of standard vaccines in childhood cancer patients.

Factors		Serology (Positive) (*N* = 76)	Serology (Negative) (*N* = 35)	*p* (Univariate)	*p* (Multivariate)	Odds Ratio	95% CI
Age (years)	Median (quartile)	12 (7–16)	6 (5–9.5)	<0.01	0.03	0.84	0.71–0.98
	Mean (SD)	11.2 (5.0)	8.1 (4.6)				
Gender	Male (%)	52 (68.4)	21 (60.0)	0.39	—	—	—
	Female (%)	24 (31.6)	14 (40.0)				
Pathology	Neural systems (%)	19 (25.0)	21 (60.0)	0.05	0.20	2.27	0.65–7.87
	NPC (%)	20 (26.3)	6 (17.1)		0.07	5.61	0.89–35.29
	Germ cells and liver (%)	14 (18.4)	0 (0.0)		0.99	< 0.01	0.00–0.01
	Sarcoma (%)	23 (30.3)	8 (22.9)		Reference	Reference	Reference
Nutrition status	Underweight (%)	14 (18.4)	14 (40.0)	0.03	0.11	2.71	0.80–9.20
	Normal for age (%)	30 (39.5)	7 (20.0)		0.28	0.50	0.14–1.79
	Overweight (%)	32 (42.1)	14 (40.0)		Reference	Reference	Reference
Chemotherapy status	Inactive (%)	60 (78.9)	31 (88.6)	0.23	—	—	—
	Active (%)	16 (21.1)	4 (11.4)				
Chemotherapy Time (years)	<0.5 years (%)	38 (50.0)	8 (22.9)	<0.01	<0.01	3.70	1.71–7.98
	0.5 to 1 year (%)	34 (44.7)	10 (28.6)				
	1–2 years (%)	4 (5.3)	17 (48.5)				
Steroid therapy	Yes (%)	57 (75.0)	28 (80.0)	0.56	—	—	—
	No (%)	19 (25.0)	7 (20.0)				
TKI use	Yes (%)	67 (88.2)	30 (85.7)	0.72	—	—	—
	No (%)	9 (11.8)	5 (14.3)				
Survival since diagnosis	Median (quartile)	29 (20.8–36)	21 (18–37)	0.21	—	—	—
	Mean (SD)	28.4 (21.5)	26.1 (22.7)				
Radiotherapy	No (%)	21 (27.6)	12 (34.3)	0.73	—	—	—
	Yes (%)	55 (72.4)	23 (65.7)				

CI, confidence interval; NPC, nasopharyngeal carcinoma; SD, standard deviation; TKI, tyrosine kinase inhibitors. +, Positive; -, Negative.

**Figure 1:**
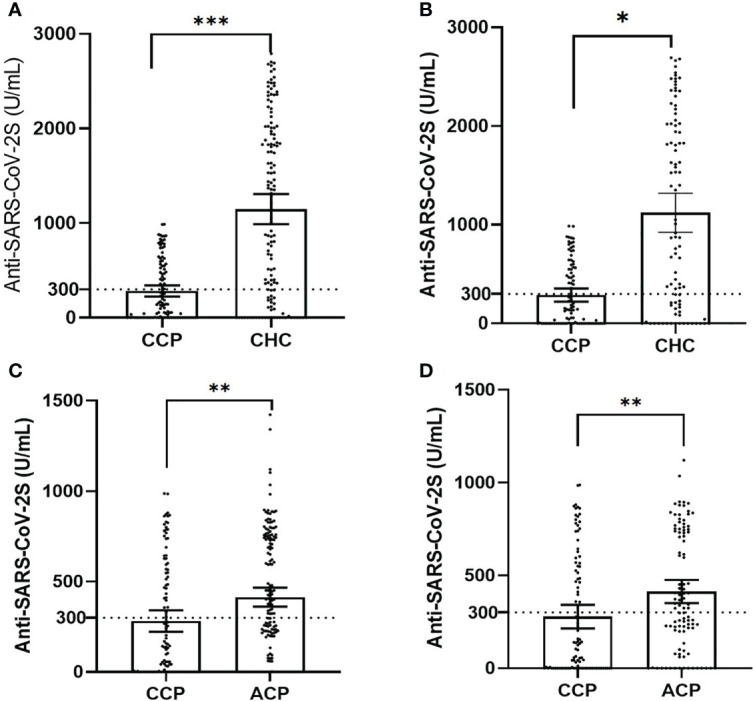
The dot plot of serology titers in unmatched and matched groups, plotted as mean ± 95% confidence intervals (CCP, childhood cancer patients; CHC, childhood healthy control; ACP, adult cancer patients; statistical power calculated to be >0.90 for all tests). **(A)** The dot plot of unmatched serology titers in the CCP versus the CHC cohort (*χ* ^2^ test, *p*< 0.001). **(B)** The dot plot of matched serology titers in the CCP versus the CHC cohort (Wilcoxon signed-rank test in 1:1 paired samples, *p* = 0.04). **(C)** The dot plot of unmatched serology titers in the CCP versus the ACP group (*χ* ^2^ test, *p*< 0.01). **(D)** The dot plot of matched serology titers in the CCP versus the ACP group (Wilcoxon signed-rank test in 1:1 paired samples, *p*< 0.01). ***p<0.001, **p<0.01, *p<0.05.

In the CHC group, 116 (86.5%) participants had positive serologic responses. Mean serologic titers were 1,210.75 ± 905.04 U/ml, and 100 (74.6%) CHC participants had an adequate response. By PSM comparison (see **Supplementary Table 2** for imbalance evaluation results), the rate of seroconversion in the CCP group was significantly less than that of the CHC group (*p*< 0.01 by paired McNemar test), and the serologic level of the CCP group was significantly less than that of the CHC group (*p*< 0.01 by Wilcoxon signed-rank test, **Figures 1A, B**). Antibody response and adverse event comparison are illustrated in **Table 3**.

**Table 3:** Propensity score-matched comparison results between CCP and CHC (*N* = 92 pairs).

Factors		CCP	CHC	p*
Efficacy Outcomes				
Serology status	Positive (%)	62 (67.4)	76 (86.7)	0.04
	Negative (%)	30 (32.6)	16 (17.3)	
Anti-S titers (U/ml)	Median (quartile)	143.0 (0.8–554.0)	897 (179.8–2021.5)	< 0.01
	Mean (SD)	287.3 (317.9)	1122.7 (954.0)	
Adequate response**	Yes (%)	36 (39.1)	62 (67.4)	< 0.01
	No (%)	56 (60.9)	30 (32.6)	
Adverse Events				
Local reactions	Yes (%)	28 (30.5)	25 (27.1)	0.76
	No (%)	64 (69.5)	67 (72.9)	
Any systemic events	Yes (%)	23 (25.0)	23 (25.0)	1.00
	No (%)	69 (75.0)	69 (75.0)	
Fever	Yes (%)	8 (8.6)	13 (14.1)	0.38
	No (%)	84 (91.4)	79 (85.9)	
Rash	Yes (%)	14 (15.2)	13 (14.1)	1.00
	No (%)	78 (84.8)	79 (85.9)	
Headache	Yes (%)	3 (3.2)	13 (14.1)	0.02
	No (%)	89 (96.8)	79 (85.9)	
Fatigue	Yes (%)	9 (9.7)	14 (15.2)	0.38
	No (%)	83 (90.3)	78 (84.8)	
Myalgia	Yes (%)	12 (13.0)	11 (11.9)	1.00
	No (%)	80 (87.0)	81 (88.1)	

*Binary categorical variables tested with McNemar test, and continuous variables tested with Wilcoxon signed-rank test. **defined as > 300 U/ml. CCP, childhood cancer patients; CHC, childhood healthy control; SD, standard deviation.

In the ACP group, 129 patients had positive serologic responses. Mean serologic titers were 414.41 ± 338.77 U/ml, and 88 patients had an adequate serologic response (53.9%). By PSM comparison between ACP and CCP groups (see **Supplementary Table 3** for imbalance evaluation results), there were significantly fewer patients in the CCP group who had positive responses than in the ACP group (*p*< 0.01 by paired McNemar test), and the titer levels were significantly lower in the CCP group as well (*p*< 0.01 by Wilcoxon signed-rank test, **Figures 1C, D**). Antibody response and adverse event comparison are illustrated in **Table 4**.

**Table 4:** Propensity score-matched comparison results between CCP and ACP (*N* = 100 pairs).

Factors		CCP	ACP	P*
Efficacy Outcomes				
Serology status	Positive (%)	67 (67)	85 (85)	0.01
	Negative (%)	33 (33)	15 (15)	
Anti-S titers (U/ml)	Median (quartile)	129 (0.7–548.5)	341 (198–739.3)	< 0.01
	Mean (SD)	278.4 (320.9)	413.2 (314.8)	
Adequate response**	Yes (%)	38 (38)	54 (54)	0.04
	No (%)	62 (62)	46 (46)	
Adverse Events				
Local reactions	Yes (%)	30 (30)	26 (26)	0.63
	No (%)	70 (70)	74 (74)	
Any systemic events	Yes (%)	25 (25)	26 (26)	1.00
	No (%)	75 (75)	74 (74)	
Fever	Yes (%)	8 (8)	12 (12)	0.50
	No (%)	92 (92)	88 (88)	
Rash	Yes (%)	17 (17)	16 (16)	1.00
	No (%)	83 (83)	84 (84)	
Headache	Yes (%)	3 (3)	10 (10)	0.09
	No (%)	97 (97)	90 (90)	
Fatigue	Yes (%)	8 (8)	14 (14)	0.26
	No (%)	92 (92)	86 (86)	
Myalgia	Yes (%)	13 (13)	18 (18)	0.44
	No (%)	87 (87)	82 (82)	

*Binary categorical variables tested with McNemar test, and continuous variables tested with Wilcoxon signed-rank test. ** defined as >300 U/ml. CCP, childhood cancer patients; ACP, adult cancer patients; SD, standard deviation.

### Neutralization of VOCs was decreased in CCP, which was associated with longer chemotherapy time

**Figure 2:**
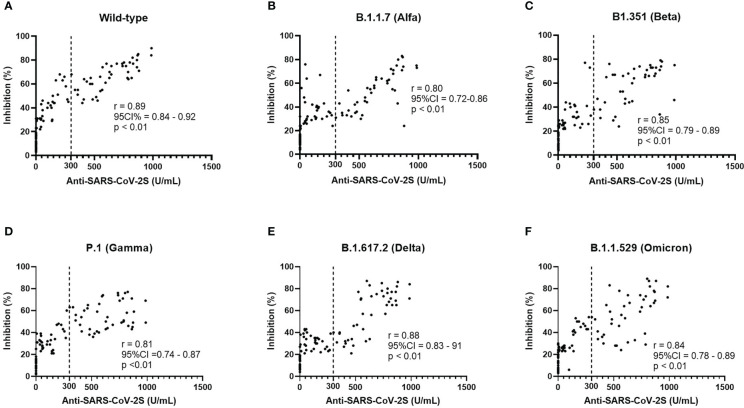
Neutralization ability **(A–F)** of different variants was found to have a positive correlation with the serologic titers of CoronaVac vaccines and the response to the wild type bears the highest level of correlation.

**Figure 3:**
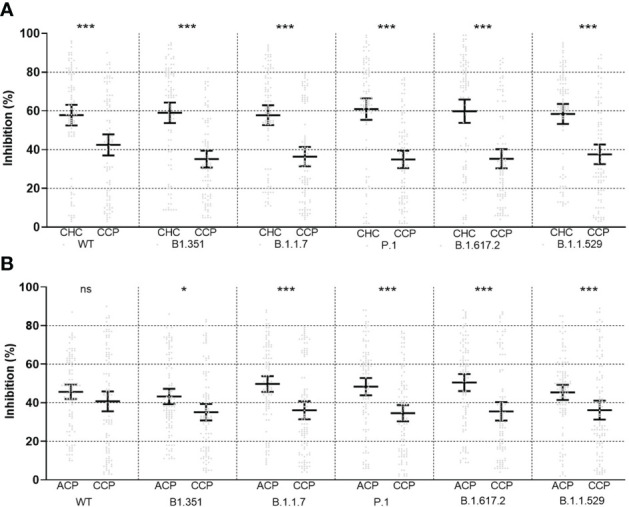
Propensity score-matched comparison of neutralizing ability between childhood healthy control (CHC, left) and childhood cancer patients (CCP, right) **(A)** and between adult cancer patients (ACP, left) and CCP (right) **(B)** after vaccination, plotted as mean ± 95% confidence intervals. ns, non-significant; *<0.05; ***<0.001. Statistical test performed with Wilcoxon signed-rank tests in 1:1 paired samples. Statistical power was calculated to be >0.90 for all tests.

Neutralization test was performed with the spike protein of wild type and VOCs. In the CCP group, percent inhibition was 41.75 ± 25.76 for wild types, 35.68 ± 21.50 for B.1.1.7 (Alfa), 36.32 ± 23.65 for B1.351 (Beta), 34.83 ± 21.13 for P.1 (Gamma), 35.93 ± 24.14 for B.1.617.2 (Delta), and 36.15 ± 24.19 for B.1.1.529 (Omicron). Analysis of variance (ANOVA) showed no significant difference among the six variants (*p* = 0.28, see **Supplementary Figure 3**). The Pearson correlation test showed that all subtypes were correlated with serologic titers of the CCP group (**Figure 2**), and the one-way ANOVA test showed no significance in neutralization ability among VOCs. Longer chemotherapy time was associated with a lower rate of inhibition for vaccines against all variants (*p*< 0.01, **Supplementary Figure 2**). In the PSM comparison, there was a significant difference in all VOCs (Alfa, Beta, Gamma, Delta, and Omicron) between the CCP and ACP group, and between the CCP and CHC group (*p*< 0.05, see **Figure 3**).

### Adverse events in unmatched groups

Solicited severe adverse events of CCP reported during follow-up were graded as I and II, and all adverse events were either self-limited or medically well-managed. Thirty-two patients had local reactions following receiving vaccines, including tenderness, rash, and itchiness. Twenty-nine patients had systemic adverse events, including fever (*n* = 9), rash (*n* = 20), headache (*n* = 3), fatigue (*n* = 11), and myalgia (*n* = 15). None of the patients had adverse events associated with solid organs or the tumor itself.

Solicited severe adverse events of the CHC group were graded as I and II and were medically managed as well. Thirty-four participants (25.3%) had local reactions, and systemic adverse events were encountered in 31 patients (23.1%). Systemic adverse events included fever (*n* = 17), rash (*n* = 16), headache (*n* = 18), fatigue (*n* = 19), and myalgia (*n* = 15).

In the retrospective ACP group, all adverse events were also graded I or II, which were medically managed. Thirty-nine patients (23.9%) reported local reactions, and systemic events were seen in 40 patients (24.5%). Systemic events included fever (*n* = 22), rash (*n* = 27), headache (*n* = 14), fatigue (*n* = 19), and myalgia (*n* = 28).

## Discussion

We evaluated the safety and immunogenicity of CoronaVac, an inactivated two-dose SARS-CoV-2 vaccine, in pediatric and adult samples of solid cancer patients, and found that vaccines were generally well tolerated without severe adverse events as compared to healthy children. We then investigated humoral response to VOCs and found that the response was moderately impaired in pediatric patients, and even more reduced as compared to adult patients, in the setting of similar adverse event profiles.

VOCs were tested in differential groups, and the vaccine exhibited differential reactive ability. Consistent with response in adult patients, response to Omicron and Delta variant was found to exhibit the lowest level of neutralization, and the result could be further validated or tested in larger-scale samples in the future. Consistent with previous research on adult patients, we found a strong correlation between serology and neutralization tests for all VOCs^7^. Although belonging to the immuno-compromised population, our study demonstrated that CCP had an even worse level of immune compromise than ACP, suggesting a prolonged period of waned response to VOCs.

Although widely rumored previously in the parents of CCP, the result confirmed that the vaccine was generally safe even in pediatric populations of altered immune status, and may strengthen healthcare messaging of inactivated vaccine promotion to the pediatric population (23–25). As hypothesized previously, the response to vaccines in pediatric populations was different from that of adult patients (26). The age range of the study was relatively young with underdeveloped levels of immune status. After long-term chemotherapy, the response to external stimuli could thus deteriorate due to the sensitivity of the immune system to chemotherapy (27, 28).

It can be noted in the analysis that although only 20 CCP (18%) were on the current regimen of chemotherapy, the therapeutic time range was still independently associated with serology failure (>1 year has the highest risk of failure). This result may suggest that the impact of chemotherapy may have added or chronological effects on vaccine responses even after cessation and was consistent with the Childhood Cancer Survivor Study, which found that significant long-term toxic effects exist for immune systems of post-chemotherapy children and young adolescents (29, 30).

Our study has several limitations. Firstly, longitudinal changes in response have yet to be reported, which will give information on the robustness of vaccine response in such populations. It should be noted that the sample size of the CCP cohort was relatively small, and the ACP group was retrospectively included to match the vaccine response of the CCP cohort. Also, we did not evaluate the cell-mediated response in our study sample, which may give further information on long-term immune response, and further studies are encouraged. These limitations can be best addressed with larger-scale, longitudinal studies and randomized trials on CCP.

## Conclusion

Humoral response against VOCs after CoronaVac vaccination in CCP was impaired although the vaccine was safe. Age and chemotherapy time seem to be reasons for poor response and low serology levels.

## Data availability statement

The raw data supporting the conclusions of this article will be made available by the authors, without undue reservation.

## Ethics statement

The studies involving human participants were reviewed and approved by the Second Affiliated Hospital of Shantou University Medical College (SAHSU). Written informed consent to participate in this study was provided by the participants’ legal guardian/next of kin.

## Author contributions

YM, BC and YQW contributed equally to this study. XJW and AZ conceptualized and designed the study, and reviewed and revised the manuscript; YM and NL designed the data collection instruments, carried out the initial analyses, and wrote the first draft of the manuscript; BC and PZ coordinated and supervised data collection and critically reviewed the manuscript for important intellectual content; YQW, ZZ, GZ, GF, DW, LC, SB, YLW, SC, JL, and XLW collected data and reviewed and revised the manuscript. All authors contributed to the article and approved the submitted version.
