# Supplementary material for: Reducedhumoral response against variants of concern in childhood solid cancer patients compared to adult patients and healthy children after SARS-CoV-2 vaccination
Source: Front Immunol. 2023 May 25;14:1110755. doi: 10.3389/fimmu.2023.1110755 (PMC10247972; doi:10.3389/fimmu.2023.1110755)
Supplement: Supplementary file 2 [file DataSheet_2.docx]

**Supplementary Materials**

**Participant Enrollment Sites**

The investigated participants were from the following hospitals: Sun Yat-sen University Cancer Center, the Second Affiliated Hospital of Shantou University Medical College, Affiliated Cancer Center of Shantou University Medical College, First Affiliated Hospital of Zhengzhou University, and Hainan Branch of General Hospital of People’s Liberation Army.

**Anti-SARS-CoV-2 binding antibody protocol**

The protocol and cut-off point to define seroconversion and methods of antibody quantification have been described previously(1).

**Neutralization Test of Variants of Concern**

The neutralization tests for variants of concerns (VOCs) were performed with a Spike RBD assay kit (Acro Biosystems, http://www.acrobiosystems.com/) through competitive ELISA methods. The microplate in the kit was pre-coated with human ACE2 protein. Positive control and Negative Control were added to the 96-test wells followed by the addition of SARS-CoV-2 Spike RBD. After incubation, the wells were washed and the substrate solution is added to the wells. The reaction was finished by the addition of stop solution to test the intensity of absorbance at 450 nm. The presence of neutralizing antibodies in the samples competed with ACE2 for SARS-CoV-2 Spike RBD binding. The intensity of the assay signal decreases proportionally with the concentration of Anti-SARS-CoV-2 neutralizing antibodies. For the current study, the ratio of dilution was 1:20 and the percent inhibition (inhibition%) was determined by 1- (OD_450nm_ of sample) / (OD_450nm_ of negative control). The negative and positive control were determined by the end user, and the cut-off inhibition% value for all kits was 20%. The catalog number of the Wild-type, Alfa, Beta, Gamma, Delta, and Omicron virus was RAS-N044, RAS-N028, RAS-N031, RAS-N034, RAS-N040, RAS-N056, respectively.

| **Supplementary Table 1. Baseline Characteristics of the Enrolled Adult Cancer Patients** | | |
| --- | --- | --- |
| **Variables** | | **Adult Cancer Patients**  **N = 163** |
| Age (years) | Median (quartile) | 51 (41-59) |
|  | Mean (SD) | 49.9 (10.7) |
| Blood drawing time since receiving vaccines (days) | Median (quartile) | 19 (17 - 22.5) |
|  | Mean (SD) | 19.75 (3.15) |
| Gender | Male (%) | 88 (54) |
|  | Female (%) | 75 (46) |
| Pathology types | GI | 42 (25.7) |
|  | NSCLC | 45 (27.6) |
|  | NPC | 76 (46.6) |
| Nutrition status | Underweight (%) | 31 (19.0) |
|  | Normal for age (%) | 57 (35.0) |
|  | Overweight (%) | 75 (46.0) |
| Chemotherapy status | Active (%) | 33 (20.3) |
|  | Inactive (%) | 130 (79.7) |
| Steroid therapy history | Yes (%) | 30 (18.5) |
|  | No (%) | 133 (81.5) |
| Chemotherapy Time (years) | < 0.5 year (%) | 47 (28.8) |
|  | 0.5 to 1 year (%) | 89 (54.6) |
|  | 1-2 years (%) | 27 (16.6) |
| Radiotherapy history | Yes (%) | 104 (63.9) |
|  | Naive or Never (%) | 59 (36.1) |
| Survival since diagnosis (months) | Median (quartile) | 26 (21 - 31) |
|  | Mean (SD) | 26.6 (19.7) |
| Notes: SD, standard deviation; GI, gastrointestinal; NSCLC, non-small cell lung cancer; NPC, nasopharyngeal carcinoma. | | |

| **Supplementary Table 2. Balance Test in Propensity Score Matching between CCP and CHC (N = 92 pairs)** | | | | | | |
| --- | --- | --- | --- | --- | --- | --- |
| **Factors** | | **Number** | | ***P**** | **Standardized Mean Difference** | |
|  |  | **CCP** | **CHC** |  | **Before matching** | **After matching** |
| Gender | Male (%) | 61 (66.4) | 59 (64.2) | 0.80 | 0.10 | 0.05 |
|  | Female (%) | 31 (33.6) | 33 (35.8) |  | -0.10 | -0.05 |
| Age | Median (quartile) | 8.0 (6.0 - 14.0) | 8.0 (6.0 - 14.3) | 0.79 | 0.07 | -0.02 |
|  | Mean (SD) | 9.7 (4.7) | 9.8 (4.6) |  |  |  |
| To compare the efficacy and adverse events between CCP and CHC groups, we carried out the PSM analysis. propensity scores were calculated that included the age and the gender of both groups, and all participants were matched in a 1:1 manner according to the score. The matching yielded 184 participants (92 pairs) with a mean propensity score of 0.45 ± 0.03. The balance test to evaluate matching performance showed that the standardized mean difference of both variables was less than the upper limit of imbalance.  Notes: *Binary categorical variables tested with McNemar test, non-binary categorical variables tested with Kappa test, and continuous variables tested with Wilcoxon signed-rank test. CCP, childhood cancer patients; CHC, childhood healthy control; SD, standard deviation. | | | | | | |

| **Supplementary Table 3. Balance Test in Propensity Score Matching between CCP and ACP (N = 100 pairs)** | | | | | | |
| --- | --- | --- | --- | --- | --- | --- |
| **Factors** | | **Number** | | ***P**** | **Standardized Mean Difference** | |
|  |  | **CCP** | **ACP** |  | **Before matching** | **After matching** |
| Gender | Male (%) | 63 (63) | 66 (66) | 0.72 | -0.25 | 0.06 |
|  | Female (%) | 37 (37) | 34 (34) |  |  |  |
| Survival since diagnosis | Median (quartile) | 25 (19.8 - 36) | 26 (22 - 31) | 0.91 | 0.13 | 0.02 |
|  | Mean (SD) | 27.2 (21.8) | 27 (19.8) |  |  |  |
| Chemotherapy time | < 0.5 year (%) | 44 (44) | 30 (30) | 0.13 | -0.14 | -0.17 |
|  | 0.5 to 1 year (%) | 36 (36) | 51 (51) |  |  |  |
|  | 1-2 years (%) | 20 (20) | 19 (19) |  |  |  |
| Steroid therapy | Yes (%) | 23 | 22 (22) | 1.00 | 0.12 | 0.02 |
|  | No (%) | 77 | 78 (78) |  |  |  |
| Nutrition status | Underweight (%) | 24 | 28 | 0.52 | 0.14 | -0.09 |
|  | Normal (%) | 35 | 34 |  | -0.03 | 0.02 |
|  | Overweight (%) | 41 | 38 |  | -0.09 | 0.06 |
| Radiotherapy | Yes (%) | 69 | 65 | 0.62 | 0.14 | 0.09 |
|  | Naive or never (%) | 31 | 35 |  |  |  |
| Chemotherapy activity | Active (%) | 19 | 19 | 1.00 | -0.06 | < 0.01 |
|  | Inactive (%) | 81 | 81 |  |  |  |
| Overall, there were 128 patients (78.5%) in the ACP group who had a positive response after vaccination, and there was a significant difference between CCP and ACP before matching (p < 0.01 by χ^2^ test). The propensity score matching yielded 200 participants (100 pairs) with a mean propensity score of 0.42 ± 0.09. The balance test showed that the standardized mean difference of each variable was less than the upper limit of imbalance.  Notes: *Binary categorical variables tested with McNemar test, non-binary categorical variables tested with Kappa test, and continuous variables tested with Wilcoxon signed-rank test. CCP, childhood cancer patients; ACP, adult cancer patients; SD, standard deviation. | | | | | | |


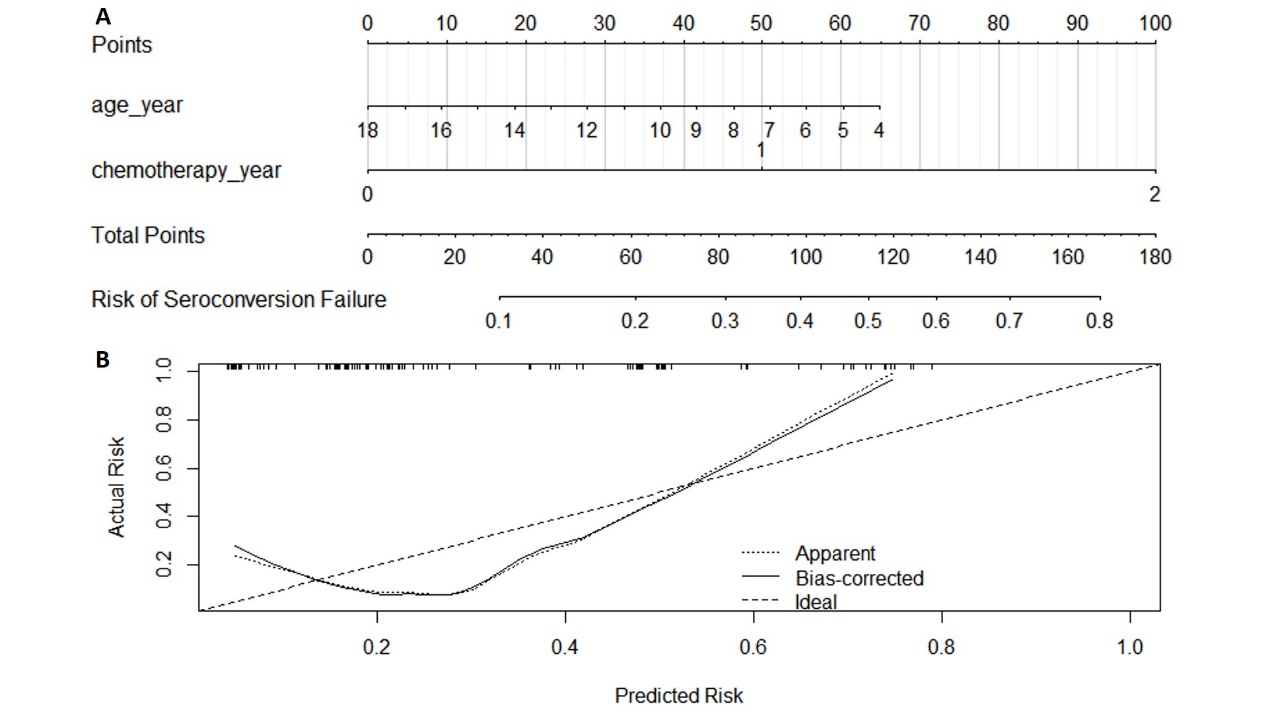


**Supplementary Figure 1.** A. A nomogram to quantify the risk of seroconversion failure after COVID-19 inactivated vaccines, which demonstrated younger age and chemotherapy time as two independent predictors. In the chemotherapy variable, “0” stands for “< half a year”, “1” stands for “between 0.5 and 1 year”, and “2” stands for “> 1 year”. B. The calibration curve of the nomogram indicates the predicted versus the actual rate of seroconversion failure.


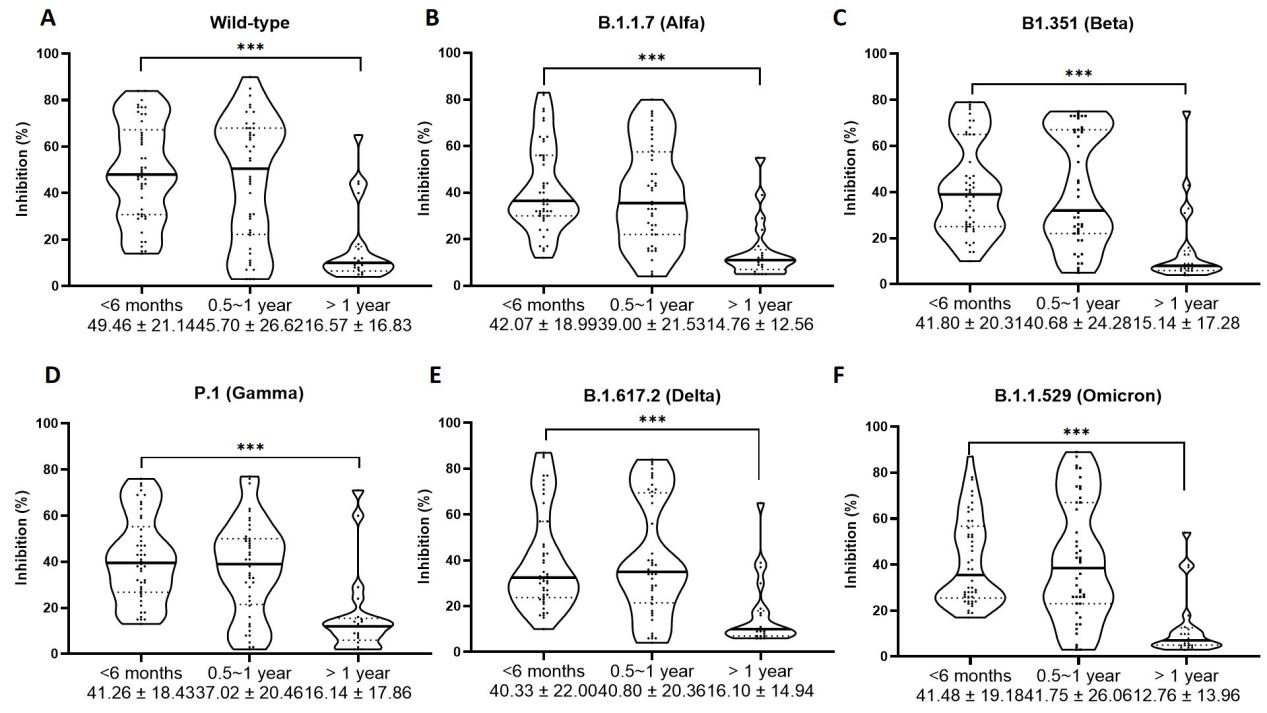


**Supplementary Figure 2.**

Neutralization ability of variants of concerns (VOCs) in the childhood cancer patients (CCP) group was divided by the chemotherapy time. The result showed that the response to all variants (Wild type, Alfa, Beta, Gamma, Delta, Omicron) was reduced in CCP who ever received chemotherapy > 1 year (A-F).


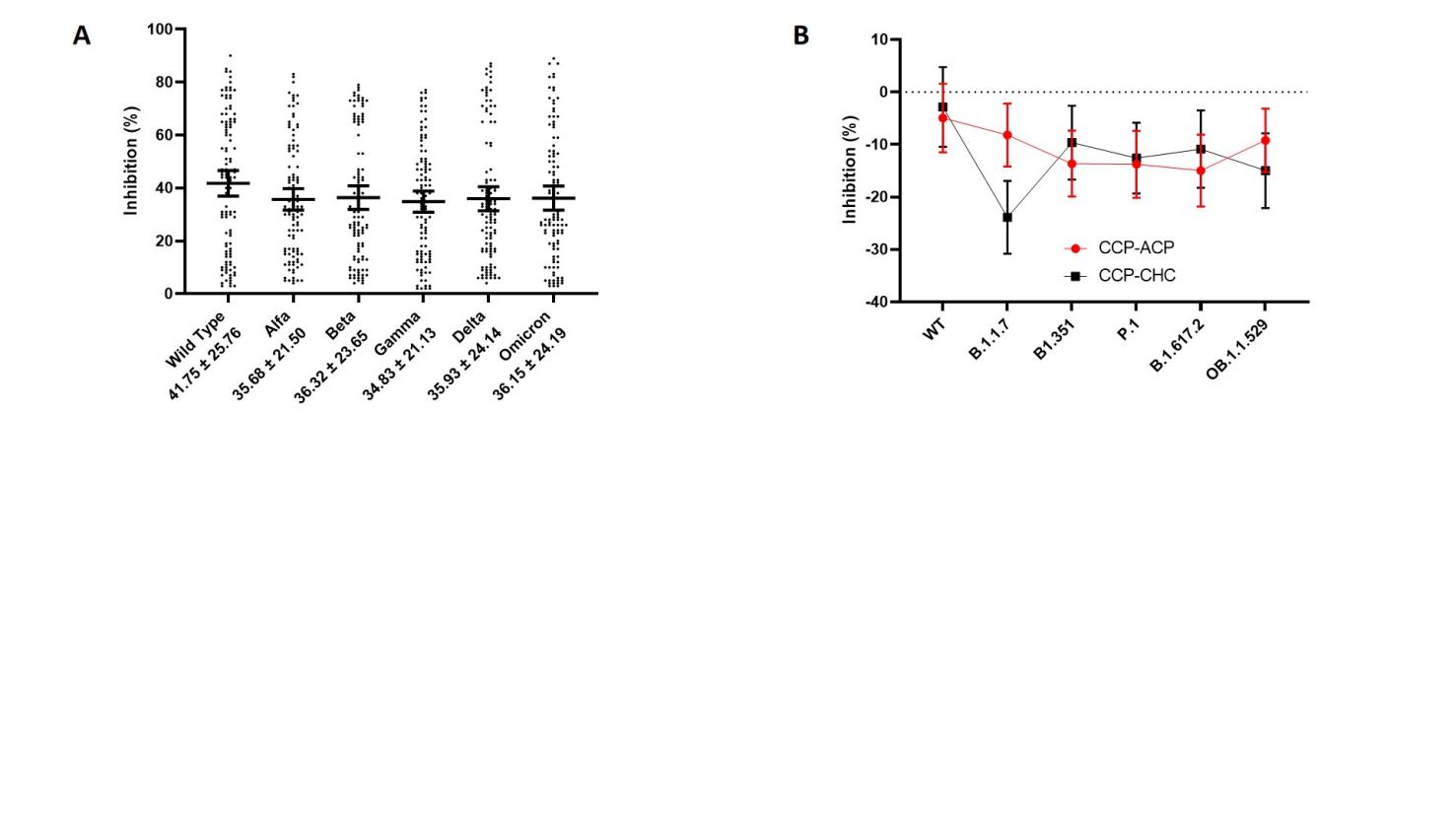


**Supplementary Figure 3.**

Neutralization ability of variants of concerns (VOCs) in the childhood cancer patients (CCP) group, and ANOVA showed no significant difference in response to all variants.

**Reference**

1. Ma Y, Liu N, Wang Y, Zeng J, Hu YY, Hao W, et al. Immune checkpoint blocking impact and nomogram prediction of COVID-19 inactivated vaccine seroconversion in patients with cancer: a propensity-score matched analysis. Journal for immunotherapy of cancer. 2021;9(11).
